# Erratum to: 1st Joint ANIRCEF-SISC Congress (meeting abstracts)

**DOI:** 10.1186/s10194-016-0709-7

**Published:** 2017-01-31

**Authors:** Paolo Martelletti

**Affiliations:** Dipartimento di Medicina Clinica e Molecolare Sapienza, University of Rome, Rome, Italy

## Erratum

Following publication of this supplement [1] it was brought to our attention that the affiliations of Dr Francesco Pierelli and Dr Ferdinando Nicoletti were incorrectly listed in a number of the abstracts published as part of this supplement [2–15].

Please note that for twelve abstracts listed [2–13] Dr Francesco Pierelli’s affiliation should be as follows:

1. Department of Medico-Surgical Sciences and Biotechnologies, Sapienza University of Rome, Rome, Italy
2. I.R.C.C.S. Neuromed, Pozzilli, Italy

Further to this, for two abstracts listed [13, 14] Dr Ferdinando Nicoletti’s affiliation should be as follows:

1. Department of Physiology and Pharmacology, University Sapienza of Rome, Rome, Italy
2. I.R.C.C.S. Neuromed, Pozzilli, Italy
